# Supplementary material for: Stress and resilience of nursing students in clinical training during political violence: A palestinian perspective
Source: PLoS One. 2025 Jun 25;20(6):e0325278. doi: 10.1371/journal.pone.0325278 (PMC12192045; doi:10.1371/journal.pone.0325278)
Supplement: S1 Supplementary Material — Scoring guidelines with normative thresholds for classification. (DOCX) [file pone.0325278.s001.docx]

**Arabic versions of the Perceived Stress Scale (PSS) and Connor-Davidson Resilience Scale (CD-RISC-10)**

**الجزء الاول: مقياس الضغط المدرك**

**(0 = غير موجود، 1 = نادرًا، 2 = أحيانًا، 3 = غالبًا، 4 = دائمًا)**

**أولًا: الضغط الناتج عن رعاية المرضى**

| **الرقم** | **البند** | **0** | **1** | **2** | **3** | **4** |
| --- | --- | --- | --- | --- | --- | --- |
| 1 | نقص الخبرة والقدرة على تقديم الرعاية واتخاذ القرارات |  |  |  |  |  |
| 2 | لا أعرف كيف أساعد المرضى في مشاكلهم النفسية والاجتماعية والجسدية |  |  |  |  |  |
| 3 | لا أستطيع تحقيق توقعاتي |  |  |  |  |  |
| 4 | لا أستطيع الرد على أسئلة الأطباء والمعلمين والمرضى |  |  |  |  |  |
| 5 | القلق من عدم ثقة المرضى أو عائلاتهم |  |  |  |  |  |
| 6 | لا أستطيع تقديم رعاية تمريضية جيدة |  |  |  |  |  |
| 7 | لا أعرف كيف أتواصل مع المرضى |  |  |  |  |  |
| 8 | أواجه صعوبة في الانتقال من دور الطالب إلى الممرض |  |  |  |  |  |

**ثانيًا: الضغط الناتج عن الواجبات وعبء العمل**

| **الرقم** | **البند** | **0** | **1** | **2** | **3** | **4** |
| --- | --- | --- | --- | --- | --- | --- |
| 1 | القلق من الحصول على درجات سيئة |  |  |  |  |  |
| 2 | الضغط بسبب طبيعة وجودة التدريب السريري |  |  |  |  |  |
| 3 | الشعور بأن الأداء لا يلبي توقعات المعلمين |  |  |  |  |  |
| 4 | متطلبات التدريب تفوق تحملي البدني والنفسي |  |  |  |  |  |
| 5 | التدريب الرتيب يؤثر على حياتي الأسرية والاجتماعية |  |  |  |  |  |

**ثالثًا: الضغط الناتج عن نقص المعرفة والمهارات**

| **الرقم** | **البند** | **0** | **1** | **2** | **3** | **4** |
| --- | --- | --- | --- | --- | --- | --- |
| 1 | لا أعرف التاريخ الطبي والمصطلحات |  |  |  |  |  |
| 2 | لا أعرف المهارات التمريضية |  |  |  |  |  |
| 3 | لا أعرف تشخيصات المرضى وعلاجاتهم |  |  |  |  |  |

**رابعًا: الضغط الناتج عن البيئة**

| **الرقم** | **البند** | **0** | **1** | **2** | **3** | **4** |
| --- | --- | --- | --- | --- | --- | --- |
| 1 | الضغط في بيئة المستشفى |  |  |  |  |  |
| 2 | عدم الألفة مع تجهيزات القسم |  |  |  |  |  |
| 3 | التوتر بسبب تغير حالة المرضى المفاجئ |  |  |  |  |  |

**خامسًا: الضغط الناتج عن الزملاء والحياة اليومية**

| **الرقم** | **البند** | **0** | **1** | **2** | **3** | **4** |
| --- | --- | --- | --- | --- | --- | --- |
| 1 | المنافسة مع الزملاء |  |  |  |  |  |
| 2 | ضغط من المعلمين الذين يقارنون الطلبة |  |  |  |  |  |
| 3 | التدريب يؤثر على الأنشطة اللامنهجية |  |  |  |  |  |
| 4 | لا أستطيع الانسجام مع زملائي |  |  |  |  |  |

**سادسًا: الضغط من المعلمين والطاقم التمريضي**

| **الرقم** | **البند** | **0** | **1** | **2** | **3** | **4** |
| --- | --- | --- | --- | --- | --- | --- |
| 1 | التباين بين النظرية والتطبيق |  |  |  |  |  |
| 2 | لا أعرف كيف أناقش حالة المرضى |  |  |  |  |  |
| 3 | المعلم يقدم تعليمات تختلف عن توقعاتي |  |  |  |  |  |
| 4 | الطاقم الطبي لا يتعاطف ولا يساعد |  |  |  |  |  |
| 5 | المعلم لا يُقيّم الطلبة بعدالة |  |  |  |  |  |
| 6 | نقص التوجيه والدعم من المعلمين |  |  |  |  |  |

**الجزء الثاني: مقياس كونور–ديفيدسون للمرونة (10 بنود)**

**(0 = غير صحيح أبدًا، 1 = نادرًا، 2 = أحيانًا، 3 = غالبًا، 4 = دائمًا)**

| **الرقم** | **العبارة** | **0** | **1** | **2** | **3** | **4** |
| --- | --- | --- | --- | --- | --- | --- |
| 1 | أتكيف مع التغيير |  |  |  |  |  |
| 2 | أتعامل مع ما يواجهني |  |  |  |  |  |
| 3 | أرى الجانب المضحك للأشياء |  |  |  |  |  |
| 4 | التوتر يجعلني أقوى |  |  |  |  |  |
| 5 | أتعافى بعد المرض أو الإصابة |  |  |  |  |  |
| 6 | أؤمن بتحقيق الأهداف رغم العقبات |  |  |  |  |  |
| 7 | أبقى مركزًا تحت الضغط |  |  |  |  |  |
| 8 | لا أستسلم بسهولة عند الفشل |  |  |  |  |  |
| 9 | أرى نفسي قويًا أمام التحديات |  |  |  |  |  |
| 10 | أتعامل مع المشاعر المزعجة |  |  |  |  |  |

**Scoring guidelines with normative thresholds for classification**

**1. Perceived Stress Scale (PSS)**

**Objective**: To measure stress levels across six domains relevant to clinical training.
**Language**: Validated Arabic version.
**Response Format**: 5-point Likert scale (0 = *Never* to 4 = *Always*).

**Subscales and Example Items**:

1. **Stress from Teachers and Nursing Staff** (6 items):
   - "I feel criticized or judged by my instructors during clinical practice."
   - "I receive insufficient feedback from my supervisors."
2. **Stress from Patient Care** (8 items):
   - "I feel anxious about making mistakes while caring for patients."
   - "Managing critically ill patients overwhelms me."
3. **Stress from Peers and Daily Life** (4 items):
   - "I struggle to balance clinical training with personal life."
   - "Competitiveness among peers adds to my stress."
4. **Stress from Assignments and Workload** (5 items):
   - "The volume of academic assignments is excessive."
   - "I have insufficient time to complete clinical tasks."
5. **Stress from the Environment** (3 items):
   - "Political instability disrupts my clinical training."
   - "Safety concerns at clinical sites increase my stress."
6. **Stress from Lack of Professional Knowledge/Skills** (3 items):
   - "I doubt my ability to perform clinical procedures correctly."
   - "I feel unprepared to handle emergency situations."

**Total Score**: Sum of all 29 items (range: 0–116).
**Classification**: Scores >75 indicate *high stress* [Sheu et al., 1997].

**2. Connor-Davidson Resilience Scale (CD-RISC-10)**

**Objective**: To assess resilience in adversity.
**Language**: Validated Arabic version.
**Response Format**: 5-point Likert scale (0 = *Not true at all* to 4 = *True nearly all the time*).

**Items**:

1. "I am able to adapt to change."
2. "I can deal with whatever comes my way."
3. "I try to see the humorous side of problems."
4. "Coping with stress strengthens me."
5. "I tend to bounce back after illness or hardship."
6. "I can achieve goals despite obstacles."
7. "I stay focused under pressure."
8. "I am not easily discouraged by failure."
9. "I think of myself as a strong person."
10. "I can handle unpleasant feelings."

**Total Score**: Sum of all 10 items (range: 0–40).
**Classification**: Scores 25–30 indicate *moderate resilience* [Campbell-Sills & Stein, 2007].

**3. Psychometric Properties**

1. **PSS**:
   - **Validity**: Content validity index (CVI = 0.94), confirmed via expert panel review.
   - **Reliability**: Cronbach’s α = 0.89 (current study), α = 0.87 (pilot study).
2. **CD-RISC-10**:
   - **Validity**: Convergent validity with psychological well-being (*r* = 0.72, *p* < 0.001).
   - **Reliability**: Cronbach’s α = 0.92 (current study), α = 0.89 (pilot study).

**4. Piloting and Refinements**

- **Sample**: 30 nursing students (excluded from the main study).
- **Adjustments**:
  - Rephrased ambiguous items (e.g., "clinical environment" → "training environment").
  - Reduced the average completion time to 12 minutes.
- **Feedback**: Participants confirmed clarity and cultural relevance of all items.
