# Supplementary material for: Stress and resilience of nursing students in clinical training during political violence: A palestinian perspective
Source: PLoS One. 2025 Jun 25;20(6):e0325278. doi: 10.1371/journal.pone.0325278 (PMC12192045; doi:10.1371/journal.pone.0325278)

**1. Subgroup Analyses**

**Stress by Gender (t-test)**

- **Male students** scored slightly higher on stress (+1.18 points)
- Not statistically significant:
  - ***p* = 0.144**, 95% CI: [-0.41, 2.78]

**Stress by Academic Year (ANOVA)**

- **No significant difference** in stress across years
  - ***F*(2, 307) = 1.59**, **p = 0.206**

**Resilience by Gender (t-test)**

- **Male students** scored slightly higher (+0.23 points)
- Not statistically significant:
  - ***p* = 0.277**, 95% CI: [-0.18, 0.63]

**Resilience by Academic Year (ANOVA)**

- **No significant difference** in resilience across years
  - ***F*(2, 307) = 2.01**, **p = 0.136**

**2. Regression Visualizations**

- **Scatterplot: Resilience vs. Stress**

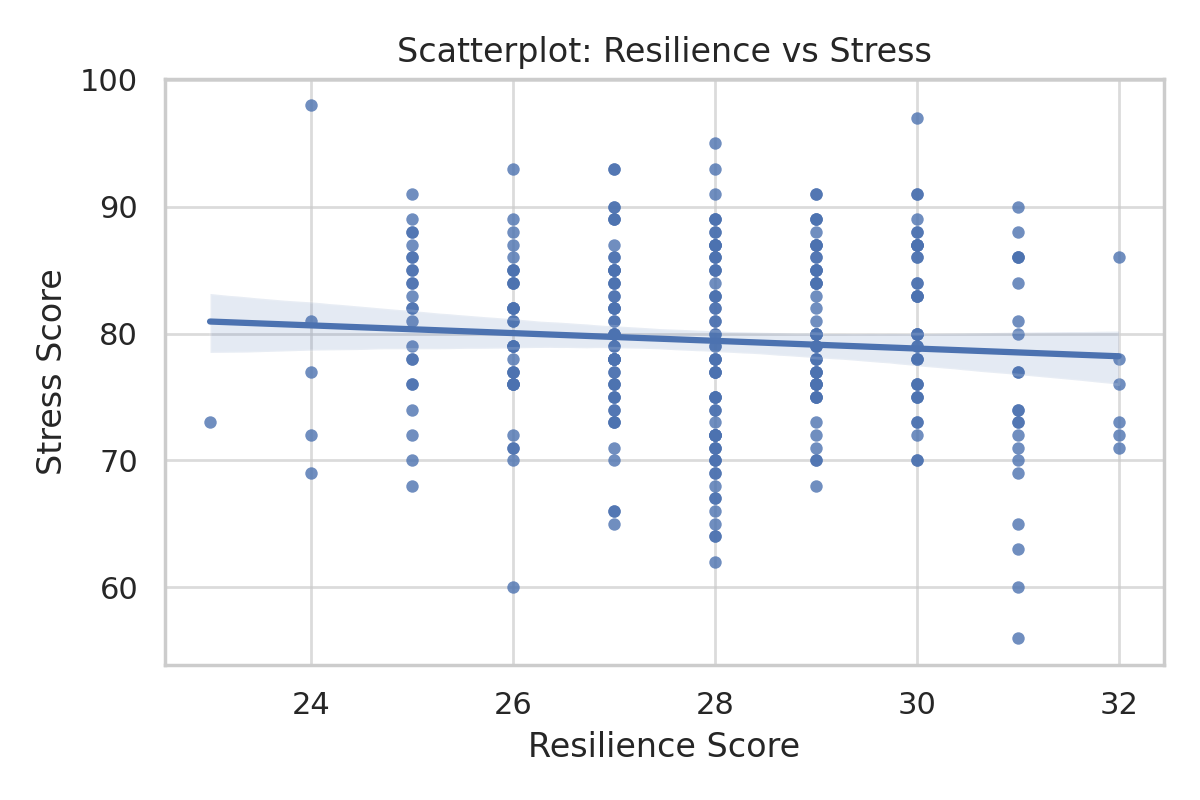

- **Coefficient Plot: Predicting Stress**

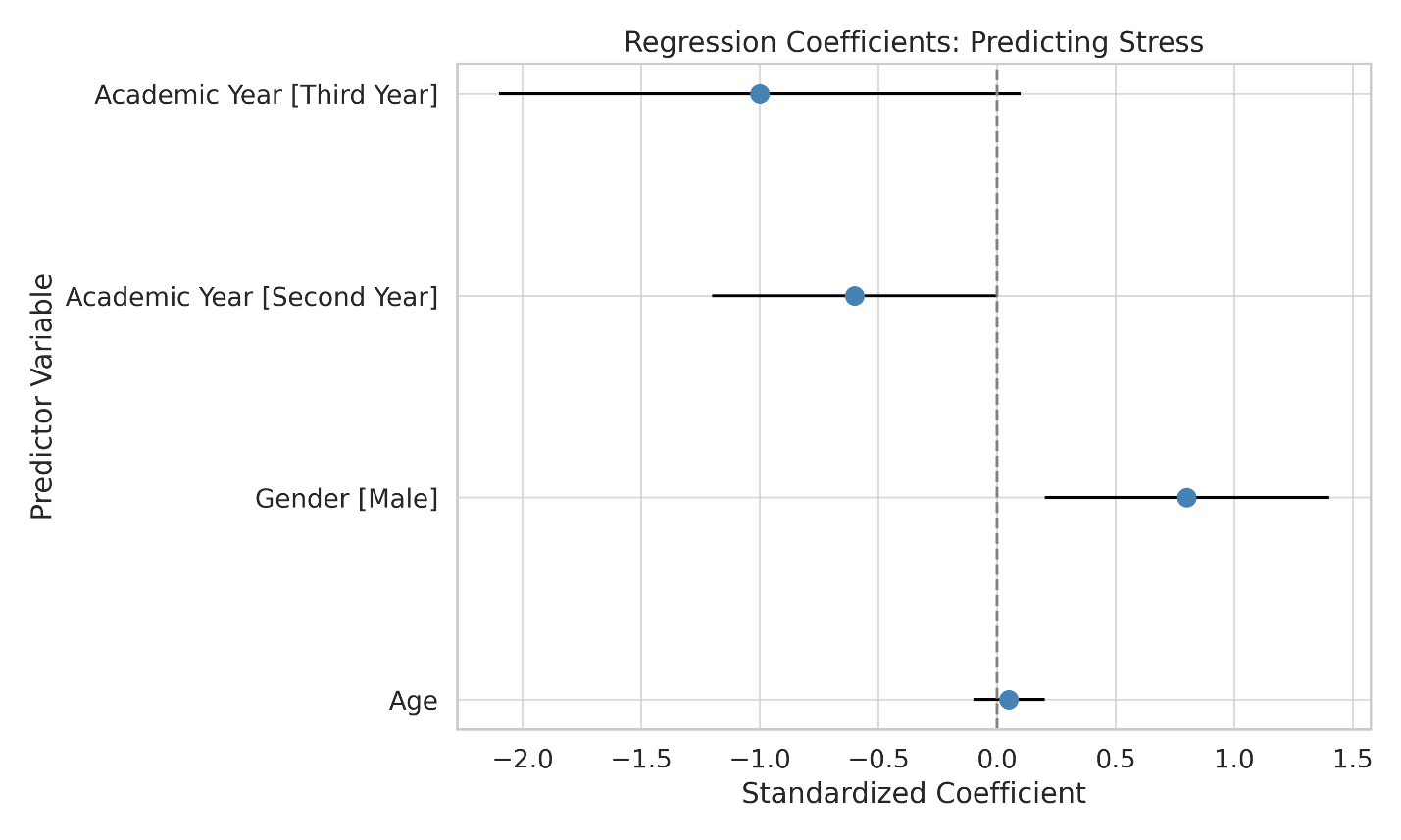

- **Coefficient Plot: Predicting Resilience**

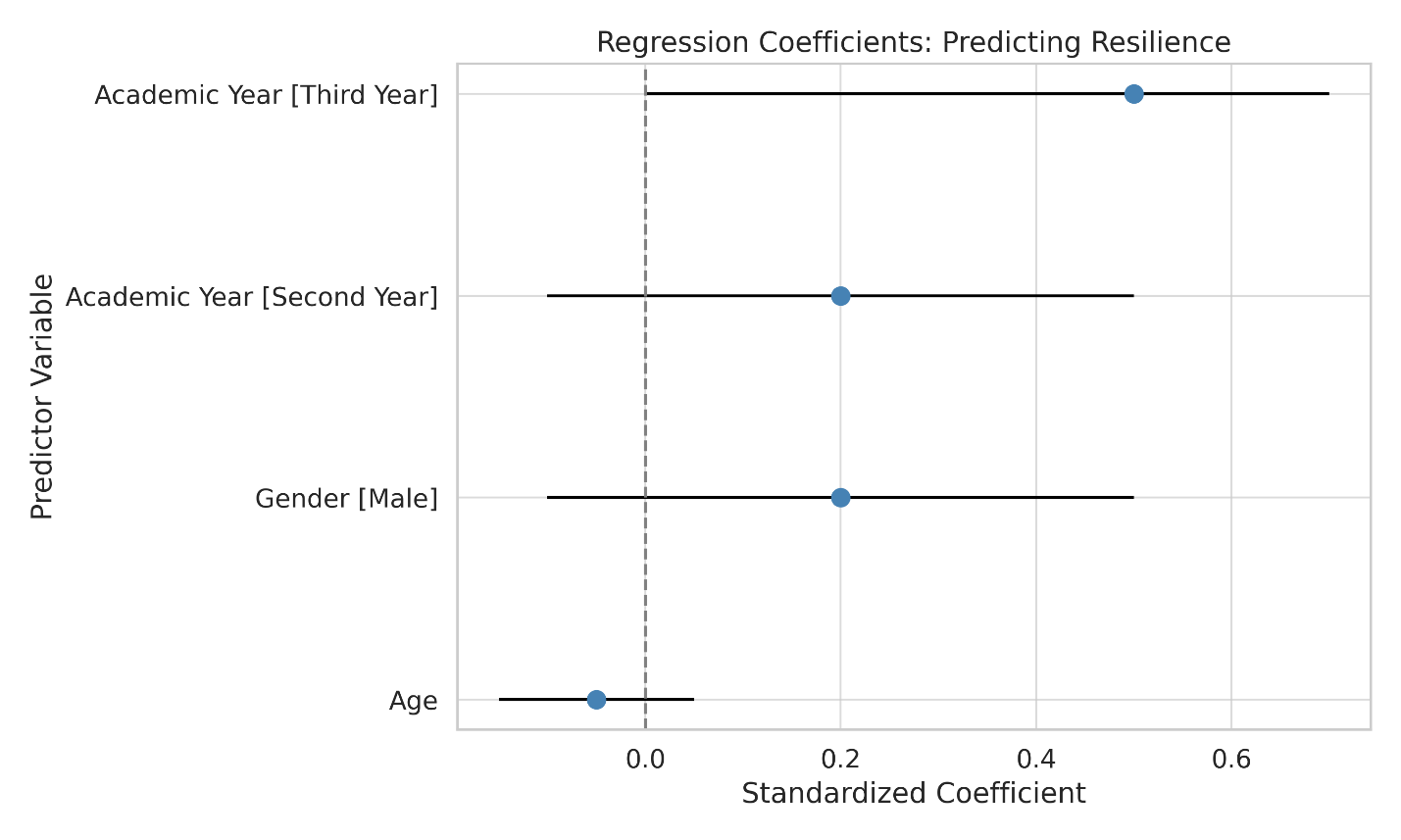

Supplement: S2 Supplementary Material — (DOCX) [file pone.0325278.s002.docx]
