# Supplementary material for: Stress and resilience of nursing students in clinical training during political violence: A palestinian perspective
Source: PLoS One. 2025 Jun 25;20(6):e0325278. doi: 10.1371/journal.pone.0325278 (PMC12192045; doi:10.1371/journal.pone.0325278)
Supplement: Attachment — Submitted filename: Response to Reviewers.pdf [file pone.0325278.s003.pdf]

Point-by-Point Responses

Reviewer #1

**Item**	**Comments**	**Response**	**Section**
**Major Concerns**			
**1. English Proofreading**	*"The Manuscript needs careful English proofreading... numerous grammar errors made this a difficult paper to read."*	Thank you for highlighting the need for language refinement. We conducted thorough English proofreading using Grammarly and a professional editor. All sections were standardized for clarity and consistency (e.g., corrected "Nursing is the most challenging undergraduate courses" → "one of the most challenging undergraduate programs").	Entire Manuscript
**2. Purpose/Objectives/Novelty**	*"The Introduction should specify the novelty of the paper compared to other works."*	We revised the Introduction to explicitly state the study's purpose, objectives, and hypothesis. The novelty (focus on nursing students in politically unstable regions, contrasting prior studies in stable environments) is now emphasized (lines 45–52).	Introduction
**3. Adjusted R^2 Values**	*"The authors must show the values of the*	Adjusted R^2 values are now included in Table 5 (Regression Analysis)	Results / Table 5

	adjusted R-square."	and discussed in the Results section (e.g., "Gender and age accounted for 3.5% of stress variance").	
**4. Tabular Results**	*"Authors must present results in tabular form and interpret them. Supplementary material was omitted."*	All results are presented in Tables 1–5, with detailed interpretations. Supplementary Tables S1–S5 (subgroup analyses, correlation matrices) are now provided.	Results / Supplementary
**5. Policy Implications**	*"The conclusions should be expanded to show policy implications."*	The Conclusion section has been expanded to outline policy implications, such as integrating resilience training into curricula and advocating for government-funded mental health initiatives in conflict zones (lines 520–535).	Discussion / Conclusion

Reviewer #2

**Item**	**Comments**	**Response**	**Section**
**Major Concerns**			
**1. English Editing**	*"Revise and re-edit the manuscript by a professional. Example: 'Three hundred and ten out of 310' → '310 out of 326.'"*	The manuscript underwent rigorous editing by a native English-speaking medical editor. Errors like "Three hundred and ten out of 310" were corrected to "310 out of 326."	Entire Manuscript

Reviewer #3

**Item**	**Comments**	**Response**	**Section**
**Major Concerns**			
**1. Validity of RQ1**	*"RQ1 lacks validity without comparison groups or standardized values. Why classify stress as 'high' and resilience as 'moderate'?"*	We now justify the "high" and "moderate" classifications using normative values from Sheu et al. (1997) and Connor & Davidson (2003). For example, PSS scores (81.1 ± 7.3) exceed normative clinical student values (72.4 ± 8.1), validating the "high" stress designation.	Results / Introduction
**2. Overreaching Conclusions**	*"Conclusions (e.g., resilience as a 'protective factor') are unsupported by the positive stress-resilience correlation."*	We revised the Discussion to align conclusions with the data. Claims about resilience as a "protective factor" were removed. Instead, we propose resilience may enable proactive engagement with stressors, leading to short-term stress.	Discussion
**3. Stress Definition**	*"The Introduction lacks a physiological definition of stress."*	A physiological definition of stress (e.g., HPA axis activation, autonomic nervous system responses) is now included in the Introduction.	Introduction
**4. Statistical Reporting**	*"Statistical parameters (e.g., effect sizes, degrees of freedom) are missing."*	All tests now report effect sizes (Cohen's d, η^2) and degrees of freedom (e.g., $t(308) = 2.20$, $p = 0.030$, Cohen's $d = 0.25$). ANOVA results include η^2 (Table 4).	Methods / Results

**5. Data Availability**	*"Raw data are not publicly available."*	The datasets are available from the corresponding author upon reasonable request due to confidentiality agreements. A summary of the data is provided in the manuscript and supplementary files.	Methods / Ethics
**Minor Concerns**			
**1. "Psychological Distress"**	*"Avoid using 'psychological distress'; use 'stress' instead."*	The term "psychological distress" was replaced with "stress" unless citing specific literature.	Terminology (Entire)
**2. Resilience as a Modulator**	*"Clarify resilience's role in modulating stressors."*	Revised to clarify: "Resilience moderates the relationship between stressors and quality of life" (line 150).	Introduction
**3. PSS/CD-RISC Citations**	*"Incorrect references for PSS and CD-RISC-10."*	References for PSS (Sheu et al., 1997) and CD-RISC-10 (Connor & Davidson, 2003) were corrected.	Methods / References
**4. Testing Procedure**	*"Provide details on session length, compensation, and recruitment steps."*	The Methods section now details session length (10–15 minutes), compensation (none), and recruitment steps (lines 210–215).	Methods
**5. α-Level**	*"Use 'α-level' instead of 'p-level'."*	"p-level" was replaced with "α-level" in the Statistical Analysis section.	Methods / Statistical
**6. Resilience Comparisons**	*"Invalid comparisons to studies using different scales."*	Comparisons to studies using different scales were removed. Scores are contextualized relative to conflict-zone populations (e.g., lines 440–445).	Discussion

**7. Sex Differences**	*"Insufficient elaboration on sex differences in stress responses."*	The Discussion now integrates literature on biological sex differences in stress responses (e.g., cortisol reactivity) alongside sociocultural factors (lines 450–460).	Discussion
**8. Sex vs. Gender**	*"Use 'sex differences' (biological) instead of 'gender differences.'"*	"Gender differences" was replaced with "sex differences" (biological categorization). Sociocultural roles are discussed separately.	Terminology (Entire)
**9. Supplementary Material**	*"Missing questionnaires and participant age ranges in supplementary material."*	Questionnaires (PSS, CD-RISC-10) and participant age ranges are now included in Supplementary Material S1.	Supplementary
**10. Stress vs. Stressors**	*"Confusion between 'stress' and 'stressors.'"*	Clarified distinctions: "stressors" (external triggers) vs. "stress" (subjective response) (lines 70–75).	Introduction / Methods
**11. Response Distribution**	*"Use 'response distribution' instead of 'response rate.'"*	"Response rate" was corrected to "response distribution" (line 200).	Methods
**12. Sample Size Typo**	*"'Three hundred and ten out of 310' → '310 out of 326.'"*	Corrected "310 out of 310" to "310 out of 326" (line 245).	Results
**13. "As such"**	*"Correct the hanging sentence: 'As such.'"*	The hanging sentence was revised (line 530).	Discussion

Reviewer #4

**Item**	**Comments**	**Response**	**Section**
**Major Concerns**			

**1. Demographic Literature**	*"Add literature on demographic factors (e.g., gender, age)."*	Literature on demographic factors (Anderson et al., 2019; Phillips et al., 2019) was added to the Introduction (lines 130–140).	Introduction
**2. Theoretical Framework**	*"State the theoretical framework guiding the study."*	The Transactional Model of Stress and Coping (Lazarus & Folkman, 1984) is now explicitly stated as the guiding theory (lines 95–100).	Introduction / Methods
**3. Inclusion/Exclusion Criteria**	*"What were the inclusion and exclusion criteria?"*	Inclusion/exclusion criteria are detailed in the Methods: e.g., excluded first-year students and those with psychiatric conditions (lines 175–180).	Methods
**4. Demographic Table**	*"Add more demographic characteristics (e.g., age)."*	Age range (19–25 years) and mean ± SD are included in Table 1.	Results / Table 1
**5. Limitation Sentence**	*"Correct the hanging sentence: 'As such.'"*	The phrase "As such" was removed from the Limitations section (line 530).	Discussion / Limitations
**6. Implications for Objective 2**	*"Add implications related to the second objective (demographic associations)."*	The Conclusion section now emphasizes tailoring interventions by sex and age (lines 540–545).	Discussion / Conclusion
