# Supplementary material for: Stress and resilience of nursing students in clinical training during political violence: A palestinian perspective
Source: PLoS One. 2025 Jun 25;20(6):e0325278. doi: 10.1371/journal.pone.0325278 (PMC12192045; doi:10.1371/journal.pone.0325278)
Supplement: Attachment — Submitted filename: Response_to_Reviewers_auresp_2.pdf [file pone.0325278.s004.pdf]

Point-by-Point Responses

Reviewer #1

**Item**	**Comments**	**Response**	**Section**
**General**	"Accept for publication because the authors took into consideration all the recommendations made by reviewer."	Thank you for your positive feedback and confirmation that all concerns were addressed.	Entire Manuscript

Reviewer #3

New Concerns

**Item**	**Comments**	**Response**	**Section**
**1. Abstract Confusion**	"Unclear if Arabic/English questionnaires were used; implies only one questionnaire."	Revised to specify that **two validated Arabic scales (PSS and CD-RISC-10)** were used, with a total completion time of 10–15 minutes.	Abstract, Methods
**2. Hypothesis Formulation**	"Hypothesis appears post-hoc; lacks backing for 'moderate resilience.'"	Added citations to prior conflict-setting studies 5,65,6 and CD-RISC-10 norms 21,2221,22 to justify the hypothesis.	Introduction
**3. Redundancy Issues**	"Duplicate sentences about supplementary materials; overlapping discussion paragraphs."	Removed duplicate references to supplementary materials and merged overlapping discussion paragraphs into a single cohesive narrative.	Discussion, Supplementary Materials

Major Concerns

**Item**	**Comments**	**Response**	**Section**
**1. Classification Justification**	"Insufficient explanation for 'moderate' resilience	Explicitly linked "moderate" resilience to 25–30 scores using norms from Pietrzak et al.	Methods, Results

	classification with CD-RISC-10."	(2009) and Campbell-Sills & Stein (2007).	
**2. Conclusion Implications**	"Still implies resilience as protective."	Revised conclusion to emphasize a **bidirectional relationship** and removed references to resilience as a "protective factor."	Conclusion
**3. Physiological Stress Background**	"Insufficient elaboration on stress physiology."	Expanded the introduction to detail chronic HPA axis dysregulation in conflict zones, citing McEwen (2017) and Yaribeygi et al. (2017).	Introduction
**4. Statistical Reporting**	"Statistical terms not italicized; methods lack clarity."	Italicized statistical terms (e.g., P, r) and restructured the Methods section to clarify analyses. Added regression model details.	Methods, Results
**5. Data Availability**	"Data availability unclear."	Supplementary Materials S1–S2 now include questionnaires, and analyses, as specified in the **Supplementary Materials** section.	Supplementary Materials

Minor Concerns

**Item**	**Comments**	**Response**	**Section**
**1. Terminology Consistency**	"Inconsistent use of 'psychological distress.'"	Replaced "psychological distress" with "stress" throughout.	Abstract, Discussion
**3. CD-RISC Citation**	"Incorrect citation for CD-RISC-10."	Corrected citation to Campbell-Sills & Stein (2007) for the 10-item scale.	Methods
**7. Sex Differences Elaboration**	"Lacks detail on biological sex differences."	Added references to HPA axis and autonomic reactivity differences 15,1615,16.	Introduction

**8. Sex vs. Gender**	"Uses 'gender' instead of 'sex.'"	Replaced "gender role expectations" with "sociocultural expectations."	Introduction
**9. Supplementary Material**	"Redundant supplementary files."	S1–S3 now contain distinct content (questionnaires, data, analyses).	Supplementary Materials
**10. Stress vs. Stressors**	"Inconsistent use of 'stress' and 'stressors.'"	Corrected instances (e.g., "stresses" → "stressors").	Discussion
**11. Response Rate**	"Incorrect use of 'response distribution.'"	Retained "response rate" for 92.2% completion.	Methods

Miscellaneous Remarks

Item	Comments	Response	Section
**Language Edits**	"First sentence in abstract is fragmented."	Revised for grammatical clarity: "Nursing is one of the most demanding undergraduate degrees..."	
