# Supplementary material for: Stress and resilience of nursing students in clinical training during political violence: A palestinian perspective
Source: PLoS One. 2025 Jun 25;20(6):e0325278. doi: 10.1371/journal.pone.0325278 (PMC12192045; doi:10.1371/journal.pone.0325278)
Supplement: Attachment — Submitted filename: Response_to_Reviewers_auresp_3.pdf [file pone.0325278.s005.pdf]

Point-by-Point Responses

Reviewer #1

Item	Comments	Response	Section
General	*"All comments have been successfully addressed... The manuscript is well-prepared for publication."*	Thank you for your positive feedback and confirmation that all methodological concerns were resolved.	Entire Manuscript

---

Reviewer #3

Item	Comments	Response	Section
1	*"The abbreviation for HPA is introduced twice in the introduction."*	Revised to ensure "hypothalamic-pituitary-adrenal (HPA) axis" is defined only once. Subsequent uses refer to "HPA axis."	Introduction
2	*"Bullet points in methods should be avoided."*	Reformatted inclusion/exclusion criteria into a bracketed paragraph without bullet points.	Methods (Study Design)
3	*"Significance indicator in Fig. 1 should more clearly show differences between subscales."*	Revised Figure 1 caption to clarify significance markers (*, $^{**, ns}$) and specify pairwise comparisons between subscales.	Results (Figure 1)
4	*"Y-axes for Fig. 1 are too narrow... They should be adjusted."*	Adjusted y-axis to range from 0–4 (based on subscale scores) and explicitly stated this in the caption	Results (Figure 1)
